# High-resolution genomic profiling and locus-specific FISH in subcutaneous and visceral adipose tissue of obese patients

**DOI:** 10.3389/fgene.2023.1323052

**Published:** 2024-03-07

**Authors:** Vivian-Pascal Brandt, Heidrun Holland, Matthias Blüher, Nora Klöting

**Affiliations:** ^1^ Saxonian Incubator for Clinical Translation (SIKT), University of Leipzig, Leipzig, Germany; ^2^ Helmholtz Institute for Metabolic, Obesity and Vascular Research (HI-MAG) of the Helmholtz Zentrum München at the University of Leipzig and University Hospital Leipzig, Leipzig, Germany; ^3^ Medical Department III–Endocrinology, Nephrology, Rheumatology, University of Leipzig Medical Center, Leipzig, Germany

**Keywords:** obesity, adiposity, visceral fat tissue, subcutaneous fat tissue, SNP array, locusspecific FISH

## Abstract

Obesity is known as a heterogeneous and multifactorial disease. The distribution of body fat is crucial for the development of metabolic complications. Comprehensive genetic analyses on different fat tissues are rare but necessary to provide more detailed information. Therefore, we performed genetic analyses of three patients with obesity using high resolution genome wide SNP array (blood, visceral fat tissue) and fluorescence *in situ* hybridization (FISH) analyses (visceral and subcutaneous fat tissue). Altogether, we identified 31 small Copy Number Variations (losses: 1p31.1, 1p22.2, 1q21.3, 2q34, 2q37.1, 3q28, 6p25.3, 7q31.33, 7q33, 8p23.3, 10q22.3, 11p15.4, 11p15.1, 11p14.2, 11p12, 13q12.3, 15q11.2-q13.1, 15q13.3, 20q13.2, 22q11.21; gains: 2q22.1-q22.2, 3p14.3, 4p16.3, 4q32.2, 6q27, 7p14.3, 7q34, 11p12, 12p11.21, 16p11.2-p11.1, 17q21.31) and 289 small copy-neutral Loss of Heterozygosity (cn-LOH). For the chromosomal region 15q11.2-q13.1, we detected a microdeletion (Prader-Willi-Syndrome) in one patient. Interestingly, we identified chromosomal SNP differences between EDTA-blood and visceral fat tissue (deletion and gain). Small losses of 7q31.33, 7q33, 11p14.2, 11p12, 13q12.3 as well as small gain of 7q34 were detected only in fat tissue and not in blood. Furthermore, FISH analyses on 7q31.33, 7q33 and 11p12 revealed differences between subcutaneous and visceral fat tissue. Generally, the deletions were detected more frequent in visceral fat tissue. Predominantly detected cn-LOH vs. CNV suggests a meaning of these cn-LOH for the pathogenesis of obesity. We conclude that the SNP array and FISH analyses used is applicable to generate more information for basic research on difficult cell subpopulations (e.g., visceral adipose tissue) and could opens up new diagnostic aspects in the field of obesity. Altogether, the significance of these mostly not yet described genetic aberrations in different fat tissues needs to confirmed in a larger series.

## 1 Introduction

Overweight and obesity are important risk factors of disease that generally concerns the health of the society and the personal health of individuals (Fontaine et al., 2003; Whitlock et al., 2009; Loos, 2018). It is becoming a growing and global epidemic in many parts of the world in developed countries (Moleres et al., 2013). The prevalence of obesity in adults has almost quadrupled during the last 4 decades. It is noticeable, that the rise in BMI has slowed down at a high level in most high-income countries. In many countries with low- and middle-income, the increase continues. Especially the global rise in obesity among children and adolescents is worrying (Loos, 2018). Obesity and overweight poses a threat to public health, linked with an exposed health risk for the development of associated disorders like type 2 diabetes, hypertension, cardiovascular disease or inflammatory disorders. Finally, the health costs are rising (Moleres et al., 2013).

Some approaches, that are aimed at prevention of obesity or promotion of weight loss due to lifestyle changes do not have an impact on community and personal levels. These insights leads to the consideration whether innate mechanisms by an encoded genome also have an impact on energy homeostasis (Loos, 2018). In addition to genetic factors, body weight is also determined by environmental factors related to lifestyle such as diet, physical activity or sedentary lifestyle and the interactions between these factors. Obesity appears to be a consequence of a positive energy balance with an alteration in one or several of these factors. With Candidate Genes Analyses and Genome Wide Association Studies (GWAS) useful tools for the detection of new polymorphisms and Copy Number Variations (CNV) associated with obesity and related comorbidities are available (Moleres et al., 2013). This has enabled scientists to identify more than 500 genetic loci linked to adiposity traits (Loos, 2018).

Adiposity is a heterogeneous disease and the development of metabolic complications is associated with fat distribution. Subcutaneous fat tissue is the natural storage of fat. Exceed of storage capacity of subcutaneous fat tissue or due to an impairment of the formation of new fat cells (genetic causes or physiologic/physical stress), so fat stores outside of the subcutaneous fat tissue (Ibrahim, 2010).

Generally, FISH analyses on visceral fat tissue are technically difficult. The aim of the current proof of concept was to implement SNP array and single cell based FISH analyses to investigate different cell subpopulation, such as EDTA-blood and visceral/subcutaneous fat tissue. In our study we used SNP array for genome-wide genetic profile analysis of very obese patients. According to current knowledge, only few studies for identification of CNV with DNA-Microarrays were performed (Thorleifsson et al., 2009; Pettersson et al., 2017). Our study revealed indications for beginning genetic instabilities. To verify incipient instabilities we performed FISH analyses. Furthermore, we have highlighted differences between EDTA-blood and visceral/subcutaneous fat tissue using SNP array. Our first results indicate that rare and small cn-LOH regions as accumulative processes could be a relevant event in the development of obesity.

## 2 Materials and methods

### 2.1 Patients

Three patients with obesity were included in the present study. The study was approved by the Ethics Committee at the University of Leipzig. Visceral (two of three patients) and subcutaneous (one of three patients) adipose tissue were removed by surgery. Detailed information on the patients (age, BMI, and diagnostic marker) are listed in Table 1.

**TABLE 1:** Clinical data including detailed information on patients age, BMI, and diagnostic marker.

Patientnumber	1	2	3	Mean ± SD
Sex	m	m	m	
Age (years)	24	26	51	33.7 ± 12.1
BMI (kg/m^2^)	69.4	47.8	77.0	64.8 ± 12.3
Body fat (%)	50.3	39.8	37.1	42.4 ± 5.6
Glucose, mmol/L	5.6	6.4	5.6	5.9 ± 0.4
Insulin, mU/L	317	47	251	205 ± 115
HbA1c (%)	5.0	11.2	8.3	8.2 ± 2.5
ALAT (µkat/L)	0.42	0.43	0.50	0.5 ± 0.03
ASAT (µkat/L)	0.39	0.44	0.45	0.4 ± 0.02
gGT (µkat/L)	0.27	0.91	0.34	0.5 ± 0.28

BMI; body mass index.

### 2.2 Molecular karyotyping using SNP array

Human fat tissue of two patients and human peripheral blood of three patients were subjected to an analysis of chromosomal regions for genome-wide CNV and identification of cn-LOH using SNP array (Affymetrix CytoScan^®^ 750 Array, ATLAS Biolabs, Berlin, Germany). Genomic DNA was isolated from blood tissue and fat tissue based on the protocols using the DNeasy Blood & Tissue Kit (50) (Qiagen, Hilden, Germany). By using agarose gel electrophoresis the DNA quality was checked. The results obtained were evaluated by using the Affymetrix Chromosome Analysis Suite (ChAS 2.0.0.195) in combination with reference data file Affymetrix CytoScanHD_Array.na33. r1. REF_MODEL, using the standard settings for CNV and cn-LOH. According to the literature, we have determined that chromosomal regions of CNV ≥3 Mb and cn-LOH ≥5 Mb are considered as reliable (Beroukhim et al., 2006; Žilina et al., 2015). Detected CNV were divided in gain [Copy Number State (CN) = 3.0] and loss [Copy Number State (CN) = 1.0]. CNV in a range of 398 kb–11 kb as well as cn-LOH regions in a range of 4,181 kb–113 kb were described as small chromosomal alterations.

### 2.3 Locus-specific fluorescence in situ hybridization

Cell preparation was carried out on EDTA-blood, visceral and subcutaneous fat tissue using standard cytogenetic techniques (colcemid treatment, hypotonic treatment and methanol/acetic acid fixation according to Seabright) (Seabright, 1972). Locus-specific FISH was performed on interphase cells in accordance to the instructions of the manufacturer’s protocol using locus-specific FISH probes from Empire Genomics, Buffalo, New York, United States. The FISH Probes RP11-997P13 (Cat. No. RP11-997P13-OR, Spectrum Orange, chromosomal region chr7: 125,871,154–126,072,115), RP11-639H21 (Cat. No. RP11-639H21-OR, Spectrum Orange, chromosomal region chr7: 133,637,186–133,823,797), and RP11-1140N3 (Cat. No. RP11-1140N3-OR, Spectrum Orange, chromosomal region chr11: 40,389,178–40,568,505) were used. Additionally, the following centromeric probes were used as internal control probes [Abbott, Chicago, Illinois, United States of America: CEP 7 (D7Z1), alpha satellite DNA, spectrum green (Cat. No. 6J3707); and CEP 11 (D11Z1), alpha satellite DNA, spectrum green (Cat. No. 6J3711)]. More than 100 interphase cells were analyzed, respectively.

For the evaluation of the FISH analyses, a cut-off level of 10% was used (related to a specific aberrant signal pattern) according to the guidelines for cytogenetic laboratory diagnostics (Leitlinien zum, 2019).

## 3 Results

### 3.1 SNP array analysis

By using the genome wide human SNP array CytoScan^®^ HD, predominantly inconspicuous results for EDTA-blood and visceral fat tissue were detected. Altogether, we identified 31 small CNV. Small gains concerned for the following chromosomal regions: 2q22.1-q22.2, 3p14.3, 4p16.3, 4q32.2, 6q27, 7p14.3, 7q34, 11p12, 12p11.21, 16p11.2-p11.1, and 17q21.31. Small losses were explored for the following chromosomal regions: 1p31.1, 1p22.2, 1q21.3, 2q34, 2q37.1, 3q28, 6p25.3, 7q31.33, 7q33, 8p23.3, 10q22.3, 11p15.4, 11p15.1, 11p14.2, 11p12, 13q12.3, 15q11.2-q13.1, 15q13.3, 20q13.2, and 22q11.21. Furthermore, 23 of these 31 CNV are not listed in Toronto-Database for Copy Number Polymorphism: 1p31.1, 1p22.2, 1q21.3, 2q34, 2q37.1, 3p14.3, 4q32.2, 6q27, 7q31.33, 7q33, 7q34, 8p23.3, 10q22.3, 11p15.4, 11p15.1, 11p14.2, 11p12, 13q12.3, 15q11.2-q13.1, 15q13.3, 17q21.31, and 20q13.2. All detailed physical positions are shown in Table 2.

**TABLE 2:** Overview of different Copy Number Variation comparing blood and fat tissue (Patient 3 without fat tissue) by SNP array.

				1		2		3
Chromosomal region	Aberration	Size (Mb)	Phys. position (Mb)	Blood	Fat tissue	Blood	Fat tissue	Blood
1p31.1	Loss	0.048	72.764.808–72.812.440	-	-	-	-	+
1p22.2	Loss	0.003	89.475.108–89.477.966	-	-	-	-	+
1q21.3	Loss	0.012	152.761.910–152.773.905	-	-	-	-	+
*2q34	Loss	0.011	213.181.329–213.192.145	-	-	+	-	-
*2q34	Loss	0.005	213.186.535–213.191.406	-	-	-	+	-
2q37.1	Loss	0.103	233.209.122–233.311.912	-	-	-	-	+
3p14.3	Gain	0.006	57.034.105–57.040.308	-	-	-	-	+
*4q32.2	Gain	0.050	161.952.213–162.002.577	-	-	+	-	-
*4q32.2	Gain	0.055	161.952.582–162.007.295	-	-	-	+	-
6q27	Gain	0.260	168.333.307–168.593.036	-	-	-	-	+
7q31.33	Loss	0.015	126.039.843–126.055.212	-	+	-	-	-
7q33	Loss	0.025	133.790.396–133.815.433	-	+	-	-	-
7q34	Gain	0.011	142.475.286–142.486.484	-	-	-	+	-
8p23.3	Loss	0.170	392.969–563.413	-	-	+	+	-
*10q22.3	Loss	0.398	81.446.906–81.844.436	-	-	-	+	-
*10q22.3	Loss	0.397	81.447.245–81.844.436	+	-	+	-	-
11p15.4	Loss	0.023	5.786.044–5.809.230	-	-	+	+	-
*11p15.1	Loss	0.021	18.941.196–18.962.398	-	+	-	-	-
*11p15.1	Loss	0.021	18.941.196–18.961.975	+	-	-	-	-
11p14.2	Loss	0.004	26.677.866–26.681.396	-	+	-	-	-
*11p12	Gain	0.080	37.754.659–37.834.730	-	-	+	-	-
*11p12	Gain	0.058	37.767.805–37.825.768	-	-	-	+	-
11p12	Loss	0.010	40.541.579–40.551.846	-	+	-	-	-
13q12.3	Loss	0.007	31.925.070–31.932.148	-	+	-	-	-
*15q11.2-q13.1	Loss	4,918	23.615.768–28.534.245	-	-	-	+	-
*15q11.2-q13.1	Loss	4,903	23.620.191–28.522.838	-	-	+	-	-
*15q13.3	Loss	0.380	32.914.238–33.294.254	-	-	+	-	-
*15q13.3	Loss	0.380	32.914.239–33.294.254	-	-	-	+	-
*17q21.31	Gain	0.080	44.212.823–44.292.742	-	-	+	+	-
*17q21.31	Gain	0.064	44.212.823–44.276.618	+	+	-	-	-
*20q13.2	Loss	0.014	52.644.753–52.658.593	-	-	-	+	-
*20q13.2	Loss	0.006	52.652.346–52.658.638	-	-	+	-	-

**FIGURE 1:**
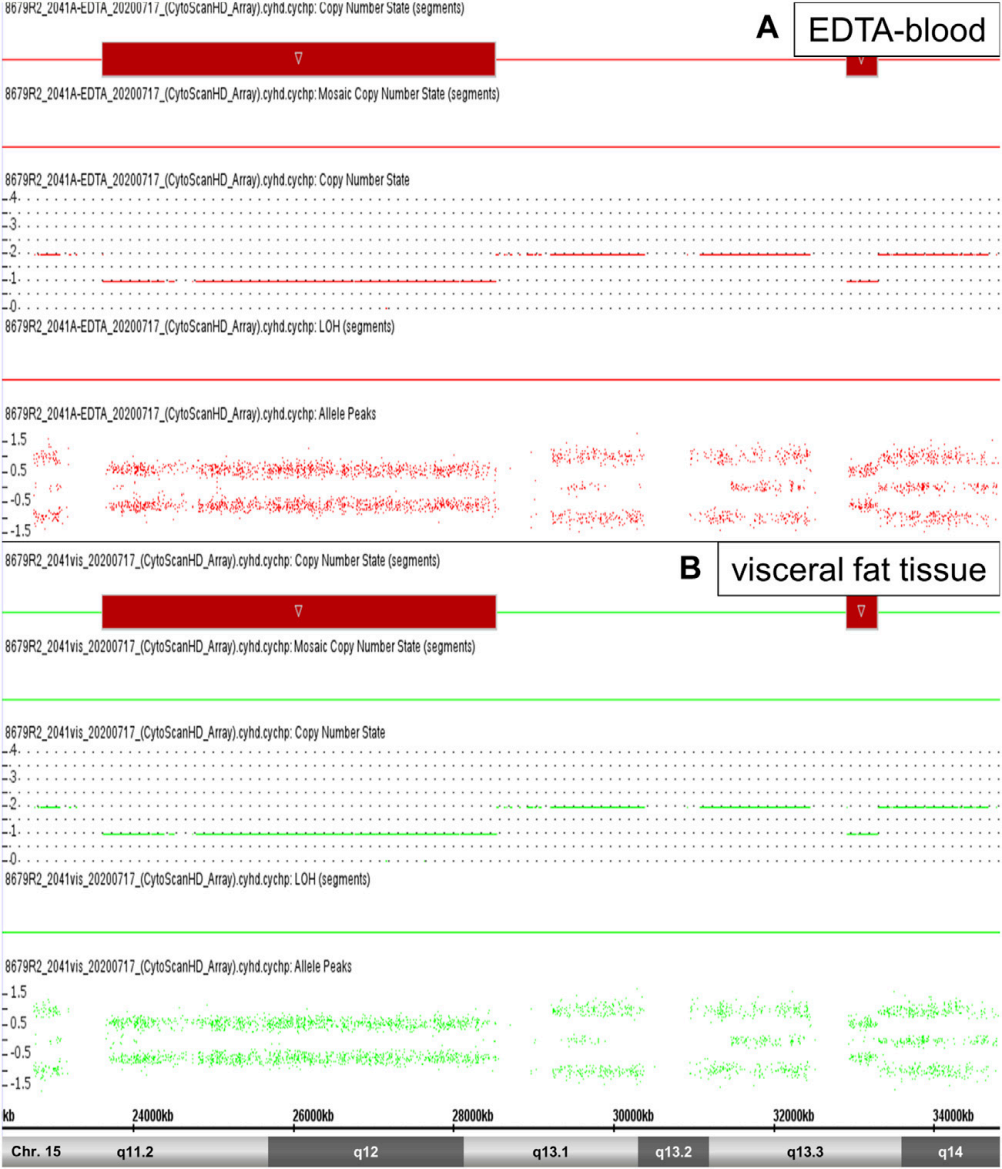
Prader-Willi-Syndrome. High-resolution Affymetrix CytoScan 750 Array analysis of the chromosomal region 15q11.2-q14 revealed an microdeletion 15q11.2-q13.1 (PWS) in patient 2 (red segments). SNP array data from EDTA-blood [**(A)**, red color)] and visceral fat tissue [**(B)**, green color)] showed this microdeletion, respectively (see Copy Number State 1,0 and Allele Peaks ~1,0).

In the current literature, CNV with a size ≥3 Mb are considered significant (Beroukhim et al., 2006)]. These criterion is fulfilled only by the chromosomal region 15q11.2-q13.1 with a length of 4.9 Mb (4,903 Mb in EDTA-blood and 4,918 Mb in fatty tissue). These microdeletion of paternal origin represents an constitutional event and is defined as Prader-Willi-Syndrome (PWS) (Figure 1). All other regions are smaller than 398 kb and therefore they were classified as small gains and losses. Chromosomal regions of interest might be 7q31.33, 7q33, 11p14.2, 11p12, 13q12.3 (small losses) as well as 7q34 (small gain) because of their detection only in fatty tissue (not detected in EDTA-blood). In addition, these regions are not described in the Toronto-Database of Genomic Variants (Table 2).

**FIGURE 2:**
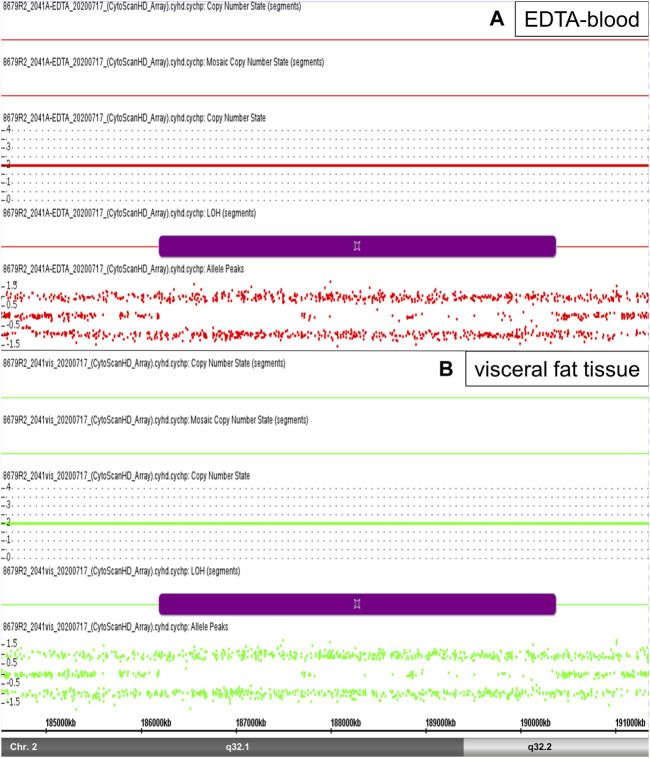
cn-LOH region. High-resolution Affymetrix CytoScan 750 Array analysis of the chromosomal region 2q32.1-32.2 revealed an cn-LOH region 2q32.1-q32.2 (size 4.18 Mb) in patient 2 (violet segments). SNP array data from EDTA-blood [**(A)**, red color)] and visceral fat tissue [**(B)**, green color)] showed this cn-LOH region, respectively (see Copy Number State 2,0 and Allele Peaks +1/-1, without value 0).

Normally, physical sequences with chromosomal aberration of cn-LOH ≥ 5 Mb are considered to be significant. A total of 289 small cn-LOH regions were found in the analyzed samples in a range of 0.113–4,181 Mb. Among these regions, we detected the largest cn-LOH aberration at chromosomal region 2q32.1-q32.2 (Figure 2). Interestingly, the most adipose patient (BMI: 77) showed the highest number of detected cn-LOH regions (95 cn-LOH regions) (Table 3).

**TABLE 3:** Overview of the detected cn-LOH regions in blood and fat tissue by SNP array (patient 3 without fat tissue).

Patient ID	BMI	cn-LOH regions^a^
**1**	69,4	fat tissue
		1p35.2-p35.1, 1p33, 1p32.3, 1q31.1, 2p11.2, 2q14.1-q14.2, 2q21.3-q22.1, 2q24.3, 2q32.3, 2q32.3-q33.1, 3p21.31, 3p21.2-p21.1, 3p12.2-p12.1, 3q21.3-q22.1, 3q26.33, 4p16.1, 4p15.31, 4p15.1, 4q22.3-q23, 4q26, 4q31.3, 5p12-p11, 6p22.1, 6p21.1, 6q22.31, 7p14.3, 7q11.21, 7q21.11-q21.12, 7q22.1, 7q31.31, 7q36.1, 8p11.21-p11.1, 8q11.1-q21.11, 8q21.2-q21.3, 8q21.3, 8q22.2, 8q24.3, 10q22.1-q22.2, 10q24.32-q25.1, 10q25.3, 11p12, 11p11.12, 11q13.4, 12q11-q12, 12q24.11-q24.13, 13q31.1, 14q12, 14q23.3-q24.1, 15q13.2-q13.3, 15q21.3, 15q23-q24.1, 16q11.2-q12.1, 16q21-q22.1, 17p11.2, 17q21.31, 17q22-q23.1, 17q23.2-q23.3, 19p12, 19q13.2-q13.31, 20q11.21, 20q11.21-q11.23
		blood
		1p35.2-p35.1, 1p33, 1p32.3, 1q31.1, 2p11.2, 2q14.1-q14.2, 2q21.3-q22.1, 2q24.3, 2q32.3, 2q32.3-q33.1, 3p21.31, 3p21.2-p21.1, 3p12.2-p12.1, 3q21.3-q22.1, 3q26.33, 4p16.1, 4p15.31, 4p15.1, 4q22.3-q23, 4q26, 4q31.3, 5p12-p11, 6p22.1, 6p21.1, 6q22.31, 7p14.3, 7q11.21, 7q21.11-q21.12, 7q22.1, 7q31.31, 7q36.1, 8p11.21-p11.1, 8q11.1-q21.11, 8q21.2-q21.3, 8q21.3, 8q22.2, 8q24.3, 10q22.1-q22.2, 10q24.32-q25.1, 10q25.3, 11p12, 11p11.12, 11q13.4, 12q11-q12, 12q24.11-q24.13, 13q31.1, 14q12, 14q23.3-q24.1, 15q13.2-q13.3, 15q21.3, 15q23-q24.1, 16q11.2-q12.1, 16q21-q22.1, 17p11.2, 17q21.31, 17q22-q23.1, 17q23.2-q23.3, 19p12, 19q13.2-q13.31, 20q11.21, 20q11.21-q11.23
**2**	47,8	fat tissue
		1p34.3, 1p34.1, 1q21.3-q22, 1q25.2, 1q32.1, 2p13.3, 2p13.2, 2p11.2, 2q12.3, 2q21.1, 2q22.3, 2q22.3-q23.1, 2q32.1-q32.2, 2q32.3, 2q34, 2q35, 3p21.31-p21.1, 3p14.2, 3p12.3, 3q21.3-q22.1, 4p15.2-p15.1, 4p15.1, 4q13.3, 4q22.3-q23, 4q25, 4q31.22-q31.23, 4q31.3, 4q32.1, 4q33-q34.1, 5p12, 5p12-p11, 5q14.3, 5q21.3-q22.1, 5q23.3-q31.1, 6q16.1, 7q11.21, 7q11.22, 7q11.23, 7q21.11, 7q31.33, 8p21.1-p12, 8p12-p11.23, 8q11.1-q11.21, 8q21.2, 8q22.2, 9p13.3, 9q33.3, 10p12.31, 10q22.1-q22.2, 11p14.1, 11q12.3-q13.1, 11q13.4, 11q22.3, 11q22.3-q23.1, 12q12, 12q13.2-q13.3, 12q21.2-q21.31, 12q21.32-q21.33, 12q24.11, 13q12.11, 13q21.1, 13q22.3, 14q23.3-q24.1, 15q15.2-q15.3, 15q21.3, 15q23-q24.1, 15q24.1-q24.2, 16q11.2-q12.1, 16q21-q22.1, 17p11.2, 17q11.2, 17q23.1-q23.2, 18q11.1-q11.2, 19q13.2, 20p11.21-p11.1, 20q11.22-q11.23, 22q13.2
		blood
		1p34.3, 1p34.1, 1q21.3-q22, 1q25.2, 1q32.1, 2p13.3, 2p13.2, 2p11.2, 2q12.3, 2q21.1, 2q22.3, 2q22.3-q23.1, 2q32.1-q32.2, 2q32.3, 2q34, 2q35, 3p21.31-p21.1, 3p14.2, 3p12.3, 3q21.3-q22.1, 4p15.2-p15.1, 4p15.1, 4q13.3, 4q22.3-q23, 4q25, 4q31.22-q31.23, 4q31.3, 4q32.1, 4q33-q34.1, 5p12, 5p12-p11, 5q14.3, 5q21.3-q22.1, 5q23.3-q31.1, 6q16.1, 7q11.21, 7q11.22, 7q11.23, 7q21.11, 7q31.33, 8p21.1-p12, 8p12-p11.23, 8q11.1-q11.21, 8q21.2, 8q22.2, 9p13.3, 9q33.3, 10p12.31, 10q22.1-q22.2, 11p14.1, 11q12.3-q13.1, 11q13.4, 11q22.3, 11q22.3-q23.1, 12q12, 12q13.2-q13.3, 12q21.2-q21.31, 12q21.32-q21.33, 12q24.11, 13q12.11, 13q21.1, 13q22.3, 14q23.3-q24.1, 15q15.2-q15.3, 15q21.3, 15q23-q24.1, 15q24.1-q24.2, 16q11.2-q12.1, 16q21-q22.1, 17p11.2, 17q11.2, 17q23.1-q23.2, 18q11.1-q11.2, 19q13.2, 20p11.21-p11.1, 20q11.22-q11.23, 22q13.2
**3**	77	blood
		1p36.11-p35.3, 1p35.2-p35.1, 1p33, 1p32.3, 1p13.2, 1p13.1-p12, 1q21.2-q21.3, 1q21.3-q22, 1q31.1, 1q44, 2q11.1-q11.2, 2q11.2, 2q21.3-q22.1, 2q32.1, 2q32.3, 3p24.3, 3p12.3, 3p12.1-p11.1, 3q22.1, 3q24, 4p15.1, 4q13.3-q21.1, 4q24, 4q25, 4q28.3, 4q33-q34.1, 5p14.3, 5p13.2, 5p13.1, 5p12, 5q13.3, 5q14.3-q15, 6p22.2-p22.1, 6p21.33-p21.32, 6q22.1-q22.31, 6q23.3, 7q11.23, 7q22.1, 7q31.1, 7q31.31, 8p22, 8p12-p11.22, 8p11.21-p11.1, 8q21.12-q21.13, 8q21.13-q21.2, 8q21.3, 8q21.3-q22.1, 8q22.2, 9q21.13, 9q22.1-q22.2, 9q33.2, 9q33.2-q33.3, 11p14.1-p13, 11p11.2-p11.12, 11q11-q12.1, 11q13.2, 11q14.1-q14.2, 12q12, 12q21.2-q21.31, 12q21.31-q21.32, 12q21.32-q21.33, 12q24.11-q24.13, 13q14.3, 13q14.3-q21.1, 13q21.31, 14q12, 14q21.1-q21.2, 14q21.3, 14q23.3-q24.1, 14q32.33, 15q13.1, 15q15.1-q15.3, 15q21.2, 15q23-q24.1, 15q24.2-q24.3, 16p12.3, 16p12.2, 16p11.2, 16p11.2-p11.1, 16q11.2-q12.1, 16q22.1, 16q22.2, 17q12, 17q21.2-q21.31, 17q22, 17q22-q23.2, 18q12.1, 18q12.2-q12.3, 20q11.21, 20q11.22-q11.23, 22q12.2-q12.3

^a^ Detected aberrant regions of blood and fat tissue of patient 1 and patient 2 are identical.

### 3.2 Locusspecific FISH

**FIGURE 3:**
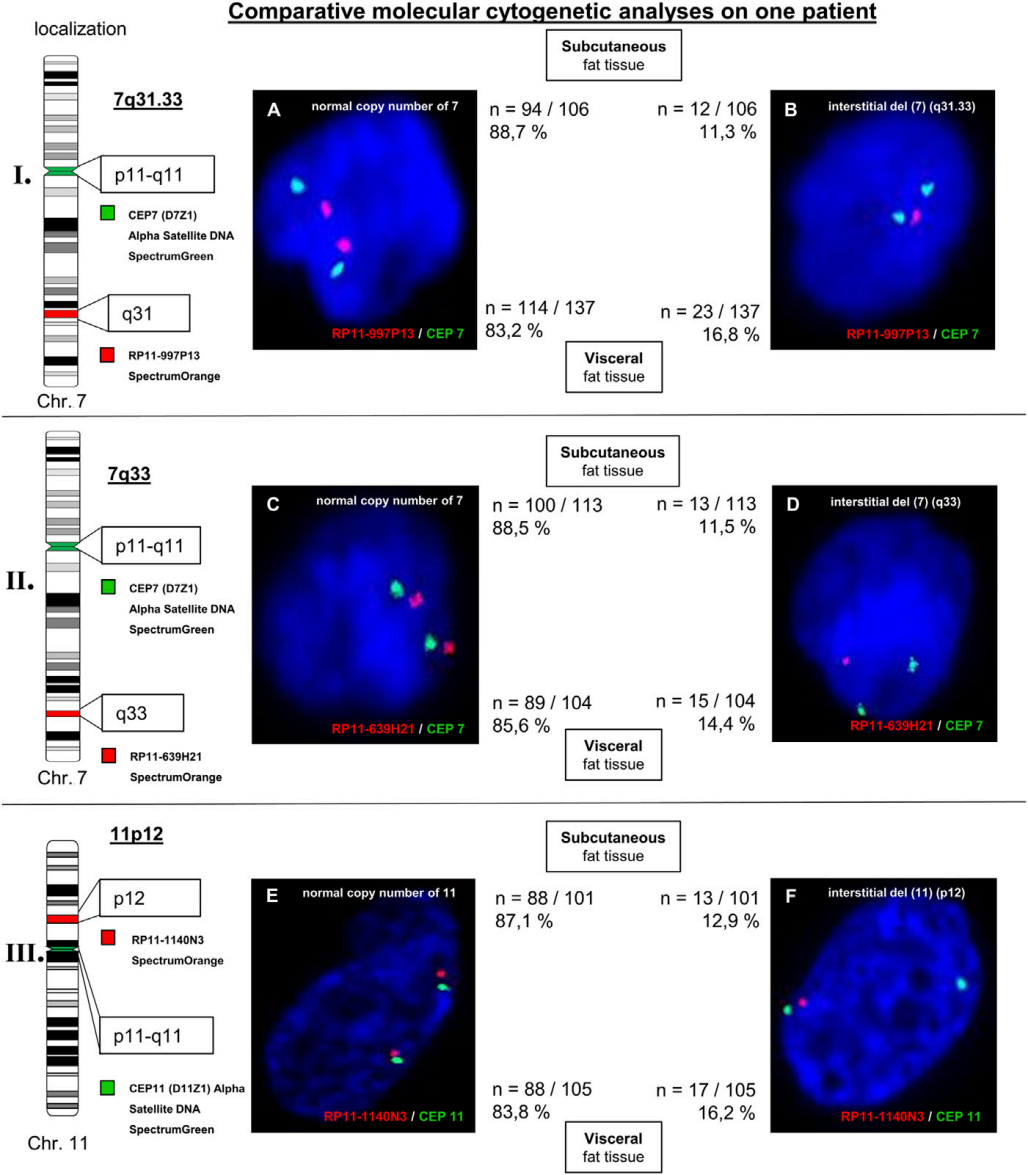
Molecular cytogenetic analyses of 7q31.33 (part I.), 7q33 (part II.), and 11p12 (part III.) on subcutaneous and visceral fat tissue of patient 1. The chromosomal ideograms on the left side display the chromosomal regions of the used FISH probes as well as the centromeric probes CEP7 and CEP11 (control FISH probes), respectively. The FISH signal patterns of analysed interphase cells are shown: normal copy numbers **(A, C)**, and **(E)** and complete interstitial deletions **(B, D)**, and **(F)**. For each signal pattern, the relative and absolute frequency of occurrence are given (subcutaneous: upper line; visceral: lower line).

For validation of possibly aberrant regions three different locusspecific FISH-probes were used. Additionally, the corresponding centromer probes [CEP 7 (D7Z1) Alpha Satellite DNA SpectrumGreen; CEP11 (D11Z1) Alpha Satellite SpectrumGreen] were used as control. An inconspicious result was detected, as ≥ 90% of the interphase nuclei do not show an aberrant signal pattern. The evaluation of the FISH technique for the choromosomal regions 7q31.33, 7q33, and 11p12 (all losses) was carried out on > 100 interphase cells. Different signal patterns were detected: interphase cells with an inconspicuous signal pattern (two signals) and complete interstitial deletion (two signals for locusspecific probes are missing). For subcutaneous fat tissue, analysed interphase cells between 11.3% and 12.9% were aberrant (for losses of 7q31.33, 7q33, and 11p12) with an conspicuous signal pattern. Different signal patterns were detected for 14,4% to 16,8% of analysed interphase cells of visceral fat tissue (losses of 7q31.33, 7q33, and 11p12; interstitital deletion). In comparison to subcutaneous fat tissue these interstitial deletions were detected more frequently in visceral fat tissue. Hybridization of centromeric probes CEP7 [(D7Z1) alpha satellite DNA SpectrumGreen] and CEP11 [(D11Z1) alpha satellite DNA SpectrumGreen] were unremarkable (Figure 3). Analyses with the database “Atlas of Genetics and Cytogentics in Oncology and Haematology” revealed two genes of interest linked to adiposity: *LRP1B* (localized at 2q22.1-q22.2) and *EXOC4* (localized at 7q33).

## 4 Discussion

In our study, we highlighted the importance of further genetic analyses in addition to Genome Wide Association Studies (GWAS). By comparing different cell subpopulations such as EDTA-blood vs. fat tissue or subcutaneous fat tissue vs. visceral fat tissue, a deeper understanding of the development of adiposity is possible. We used SNP array and locus-specific FISH to identify small genome wide differences of various cell subpopulation.

Generally, we have identified genetic aberrations which are described in the literature for adiposity. We detected a microdeletion 15q11.2-q13.1 (PWS). The PWS is defined as constitutional aberration (e.g., microdeletion) and known as a genetic cause for the development of obesity. Special, hypothalamic dysfunction is associated with hypoactivity and insatiable hunger that results in obesity (Noordam et al., 2021).

Furthermore we detected 20/23 genomic variations, which are not listed in Toronto Database for Genomic Variants, but described in context with adiposity. Our study revealed a loss of 2q37.1 in EDTA-blood. D’Angelo et al. (2018) detected CNV in 279 patients with a syndromic obesity phenotype. The authors suggest that the deletions of 2q37 cause obesity in addition to other aberrations (D’Angelo et al., 2018). Moreover we identified chromosomal abnormalities which have been rarely described in the literature. By analyzing the data, we identified a loss of the chromosomal region 3q28. The study of Rasmussen-Torvik et al. (2009) revealed an correlation between adiponectin measurements by ELISA and SNPs at the chromosomal region 3q28 by using the Marshfield Mammalian Genotyping Service Screening set 12 and GeneMapper^®^ software. Adiponectin is known in context with obesity and insulin resistance (Rasmussen-Torvik et al., 2009). Furthermore, our study revealed a small loss of the chromosomal region 20q13.2. This chromosomal region showed an evidence for the linkage for plasma adiponectin levels on chromosome 20q13.2 according to the study of May-Ruchat et al. (2008) (Ruchat et al., 2008). According to the studies of Lee et al. (1999) and Lembertas et al. (1997) this region is located in an obesity-related chromosomal region (Lembertas et al., 1997; Lee et al., 1999).

To the best of our knowledge, 3/23 detected small aberrant chromosomal regions in our study are not described in adiposity (1q21.3, 3p14.3, and 7q31.33). Most of these chromosomal regions are described in tumors, like breast cancer or brain tumors (Nagahata et al., 2002; Holland et al., 2012). Some of the chromosomal regions are associated with constitutional microdeletions, like childhood myelodysplastic syndromes (3p14.3) or intellectual disability, microcephaly, and epilepsy (1q21.3) (Praulich et al., 2010; Sonmez et al., 2017).

Interestingly, some aberrant chromosomal aberrations were detected only on fat tissue, but not on EDTA-blood: 7q31.33 (not described for obesity), 7q33, 7q34, 11p14.2, 11p12, and 13q12.3. Aberrations for the chromosomal region 7q31.33 are associated with hairy cell leukemia and splenic marginal zone lymphoma (loss of a 11.4 Mb region within the region 7q31.33 to 7q33) (Andersen et al., 2004). However, most chromosomal regions are described in context with adiposity, for example, 7q33. Within the chromosomal region 7q33, the *EXOC4*-gene is localized. The protein is involved in glucose transport into muscle and fat tissue. Further analyses with locusspecific FISH technique on three chromosomal regions (7q31.33, 7q33, and 11p12) revealed interstitial deletions. We believe, that these beginning instabilities can influence transformation processes in patients with obesity. Similar processes are described in other cell entities like tumors or arthritis (Kinne et al., 2001; Frydrychowicz et al., 2015). To the best of our knowledge, no studies are known comparing the genomic profile of subcutaneous and visceral fat tissue using SNP array (Affymetrix CytoScan 750) and locus-specific FISH analyses. The study of Linder et al. (2004) revealed an differently expressed gene pattern of subcutaneous and visceral adipose tissue of obese patients (Linder et al., 2004). Using FISH technique, our results showed a difference of subcutaneous fat tissue compared with visceral fat tissue at the genetic level (more frequent occurrence of interstitial deletion in visceral fat tissue compared with subcutaneous fat tissue). Therefore, we assume a significance of the increased genetic instabilities within the genome and the more unfavorable properties of the visceral fat tissue vs. subcutaneous fat tissue.

Additionally, we have identified a total of 289 small copy-neutral losses of heterozygosity (<5 Mb) by using high-resolution SNP array. A recent study by Loh et al. (2020) have highlighted the significance of small cn-LOH aberration in context with clonally expanded cells and stem cells. By somatic cn-LOH mutations, inherited alleles become homozygous. The rare coding variants have an impact on the development of blood clones. Alleles with cn-LOH segments promoting the expansion of hematopoetic cells leads to an increased polygenic drive for blood-cell proliferation (Loh et al., 2020). To get information about potentially affected gene expression of the 289 detected cn-LOH regions, we have carried out a comparative analysis with the Atlas of genetics and cytogenetics in oncology and haematology (Huret et al., 2013). We found 71 genes (within 289 cn-LOH regions) linked with adiposity. As an example, the gene ADIPOR1 (within the detected cn-LOH region 1q32.1) encodes for the adiponectin receptor 1 (AdipoR1) and mediates the biological responses to adiponectin (adipocyte–derived abundant plasma protein) (Lustig et al., 2012). Adiponectin is associated with obesity and insulin resistance/type-2 diabetes (Kim et al., 2006; Yamauchi and Kadowaki, 2013).

A total of 24/289 detected cn-LOH regions are larger than 2 Mb. Hereby 20/24 cn-LOH regions are linked with obesity in the literature. For example, we identified an cn-LOH aberration for the chromosomal region 6q22.1-q22.31. According to the study of Meyre et al. (2004), a significant association of the chromosomal region 6q22.31-q23.2 and childhood obesity was revealed (Meyre et al., 2004).

Moreover, for 4/24 cn-LOH regions, no studies related to obesity are known in the literature (localized at 3p12.3, 7q11.21, and 8q11.1-q11.21). Aberrations within these regions are associated with different tumor entities (like adenocarcinoma or prostate cancer), delayed development disorders, or intellectual disability (e.g., schizophrenia) (Wolf et al., 2004; Maiti et al., 2011; Wang et al., 2015; Soueid et al., 2016). Furthermore, we identified the most cn-LOH regions on the sample material of the patient with the highest BMI (BMI: 77; cn-LOH regions: 95; Table 3).

Therefore, cn-LOH may have an potential impact on the further process of adiposity.

In summary, we could confirm for obesity described chromosomal aberrations (PWS) and identified not previously described small genetic aberrations, e.g., in different cell subpopulations (visceral and subcutaneous fat tissue).

Our results suggests, that an accumulation of small cn-LOH regions may have an impact on the pathogenesis of adiposity. We highlighted the importance of further analyses in addition to Candidate Genes Analyses and Genome Wide Association Studies (GWAS). SNP array and FISH analyses are appropriate genetic techniques for detecting potentially minor differences in the genetic profile even in the challenging cell subpopulations of subcutaneous and visceral fat tissue. Therefore, these comparative analyses of different cell subpopulation could provide additional information in the genetic profile on EDTA-blood vs. fat tissue and subcutaneous fat tissue vs. visceral fat tissue. Due to a small cohort and different BMI values, further investigations in a larger cohort comparing the genome-wide profile of subcutaneous and visceral adipose tissue are useful to get deeper insights into the pathogenesis of adiposity and for a better understanding of significance of these cn-LOH regions.

## Data Availability

The original contributions presented in the study are publicly available. This data can be found here: https://www.ncbi.nlm.nih.gov/dbvar/, accession number: nstd237.
